# Prognostic significance of risk stratification in CHAARTED and LATITUDE studies among Japanese men with castration-resistant prostate cancer

**DOI:** 10.1016/j.prnil.2022.01.001

**Published:** 2022-01-11

**Authors:** Sotaro Chikamatsu, Masaki Shiota, Shigetomo Yamada, Leandro Blas, Takashi Matsumoto, Eiji Kashiwagi, Junichi Inokuchi, Ken-ichiro Shiga, Akira Yokomizo, Masatoshi Eto

**Affiliations:** a Department of Urology, Graduate School of Medical Sciences, Kyushu University, Fukuoka, Japan; b Department of Urology, Harasanshin Hospital, Fukuoka, Japan

**Keywords:** Androgen-deprivation therapy, Castration-resistant prostate cancer, Docetaxel, Androgen receptor pathway inhibitor, Risk stratification

## Abstract

**Background:**

The CHAARTED and LATITUDE trials demonstrated a survival benefit of docetaxel and abiraterone for hormone-sensitive prostate cancer. In this study, we examined the impact of the risk stratification criteria used in the CHAARTED and LATITUDE trials on the prognosis of castration-resistant prostate cancer (CRPC). We also tested whether these risk stratification criteria could help in selecting effective initial treatment for CRPC.

**Method:**

Japanese patients with CRPC who were treated with docetaxel or androgen receptor pathway inhibitors such as abiraterone acetate or enzalutamide between 2014 and 2018 were included in this study. Clinicopathological factors, progression-free survival, and overall survival were investigated.

**Results:**

Of 215 patients, 110 men (51.2%) and 93 men (43.3%) were grouped as high volume by CHAARTED criteria and high risk by LATITUDE criteria, respectively. Median progression-free survival was 10.3/4.5 months (*P* < 0.0001) for low/high volume (CHAARTED criteria) and 9.9/4.8 months (*P* = 0.0032) for low/high risk (LATITUDE criteria). The median overall survival was 44.8/17.4 months (*P* < 0.0001) for low/high volume (CHAARTED criteria) and 37.4/17.4 months (*P* = 0.0011) for low/high risk (LATITUDE criteria). The prognostic impact of CHAARTED and LATITUDE criteria was comparable between androgen receptor pathway inhibitors and docetaxel as first-line treatment for CRPC.

**Conclusion:**

The CHAARTED and LATITUDE criteria were prognostic, but not useful to discriminate the therapeutic outcome between androgen receptor pathway inhibitors and docetaxel for CRPC.

## 1 Introduction

Androgen-deprivation therapy (ADT), which suppress androgen production and inhibits androgen activity, has been the standard treatment for recurrent or advanced prostate cancer since 1941.^1^ However, most recurrent and advanced prostate cancers are not cured by ADT and relapse as metastatic castration-resistant prostate cancer (CRPC).^2^ Docetaxel and androgen receptor pathway inhibitors (ARPIs), such as abiraterone acetate and enzalutamide, are first-line agents for CRPC and prolong survival.^3^, ^4^, ^5^, ^6^, ^7^ Therefore, docetaxel chemotherapy and ARPIs have become one of the standard treatments for metastatic CRPC.^8^

Interestingly, in metastatic hormone-sensitive prostate cancer (HSPC), the survival benefits of these therapies may vary depending on tumor aggressiveness, tumor burden, and tumor spread. In the CHAARTED trial, up-front docetaxel chemotherapy combined with ADT showed a significant survival benefit in the high-volume group (defined as having visceral metastases, or 4 or more bone metastases beyond the spine and pelvis), but not yet in the low-volume group.^9^ Similarly, in the LATITUDE trial, there was a significant survival benefit in the high-risk group (defined as having at least two of [a] Gleason score of 8 or higher, [b] bone metastases of 3 or more, or [c] visceral metastases) when treated with up-front abiraterone acetate.^10^

These criteria have also been reported to be prognostic factors for HSPC patients treated with ADT.^11^ On the other hand, it has been reported that risk classification by CHAARTED criteria at the time of initial diagnosis of HSPC is not significantly associated with overall survival (OS) after CRPC progression.^12^ However, the prognosis of CRPC stratified by these criteria at the time of diagnosis of CRPC has not been shown. Therefore, we investigated whether the LATITUDE and CHAARTED criteria at the time of CRPC diagnosis could be prognostic factors in the primary treatment of CRPC. We also investigated whether these criteria could be predictors of response to treatment with ARPI or docetaxel for CRPC.

## 2 Materials and methods

### 2.1 Patients

In this study, Japanese men who received primary treatment with ARPI (abiraterone acetate or enzalutamide) or docetaxel for CRPC at Kyushu University Hospital (Fukuoka, Japan) and Harasanshin Hospital (Fukuoka, Japan) from May 2014 to December 2018 were retrospectively enrolled. The study was approved by the respective institutional review boards. All patients were histopathologically diagnosed with adenocarcinoma of the prostate and underwent ADT. Clinical stage was determined using the uniform TNM criteria based on the results of digital rectal examination, transrectal ultrasonography, magnetic resonance imaging, computed tomography, and bone scintigraphy.^13^ CRPC was diagnosed in patients with increased prostate-specific antigen (PSA) levels (> 2 ng/mL and 25% increase) and/or radiographic progression despite ADT by the physician's judgment.^14^

### 2.2 Treatment

ARPI with either abiraterone (1,000 mg/day) and prednisolone (10 mg/day), or enzalutamide (160 mg/day) was administered as reported previously.^4^, ^5^, ^6^, ^7^ Docetaxel was administered using a 3- or 4-weekly (70–75 mg/m^2^) regimen as reported previously.^15^^,^^16^ During treatment with ARPI or docetaxel, castration status was maintained by surgical or continuous medical castration with a luteinizing hormone-releasing hormone agonist (goserelin acetate or leuprorelin acetate) or antagonist (degarelix acetate). Treatment with ARPI or docetaxel was continued at the physician's discretion based on disease progression, adverse events, or patient refusal.

### 2.3 Endpoints

Risk stratification was assessed at the time of diagnosis of CRPC according to the criteria used in the CHAARTED and LATITUDE trials. Disease progression was assessed by PSA increase of > 2 ng/mL and 50% increase over the nadir, or radiographic progression defined as the appearance of two new lesions or progression of one or more known lesions, as classified by the response evaluation criteria in solid tumors (RECIST).^14^

### 2.4 Statistical analysis

All statistical analyses were performed using JMP14 software (SAS Institute, Cary, NC, USA). Continuous and categorical data were compared by Wilcoxon's rank sum and Pearson's chi-square test, respectively. Survival analysis was performed using the Kaplan–Meier method and the log-rank test. Cox proportional hazards model was used to estimate the hazard ratio (HR). Differences in the prognostic impact of subgroups were examined by interaction tests. All tests were two-sided, and *P* < 0.05 was considered significant.

## 3 Results

A summary of the characteristics in 215 patients is shown in Table 1. The median age of the patients was 74 years (interquartile range [IQR], 69–81 years), and the median PSA at the onset of CRPC was 15.7 ng/ml (IQR, 6.3–62.9 ng/ml). Forty-five patients (20.9%) were non-metastatic at CRPC. When classified using the risk stratification of the CHAARTED and LATITUDE studies, 110 patients (51.2%) and 93 patients (43.3%) were judged to be high volume by the CHAARTED criteria and high risk by the LATITUDE criteria, respectively. About two-thirds of the patients had a Gleason score of 8 or higher, and most had bone metastases, but less than one in ten had visceral metastases. The median time from first treatment to CRPC was 15.6 months (IQR, 8.0–29.1 months). As primary treatment for CRPC, 162 men received ARPI [abiraterone in 57 patients (26.5%); enzalutamide in 105 patients (48.8%)] while 53 patients (24.7%) were treated with docetaxel. During a median follow-up of 19.3 months (IQR, 8.8–32.5 months), 177 patients (82.3%) experienced disease progression, and 120 patients (55.8%) died from any cause.

**Table 1:** Patients' characteristics

	All (*n* = 215)
Median age, years (IQR)	74 (69–81)
Median PSA, ng/mL (IQR)	15.7 (6.3–62.9)
Median time to CRPC, months (IQR)	15.6 (8.0–29.1)
Gleason score, n (%)	
≤ 8	72 (33.4%)
> 8	137 (65.6%)
Not available	6
Prior local treatment, *n* (%)	
Absence	146 (67.9%)
Presence	69 (32.1%)
Surgery	26 (12.1%)
Radiation	43 (20.0%)
Bone metastasis, *n* (%)	
Presence	152 (70.7%)
Absence	63 (29.3%)
Visceral metastasis, *n* (%)	
Presence	19 (8.8%)
Absence	196 (91.2%)
CHAARTED criteria, *n* (%)	
Low volume	105 (48.8%)
High volume	110 (51.2%)
LATITUDE criteria, *n* (%)	
Low risk	115 (55.3%)
High risk	93 (44.7%)
Not available	7
First-line treatment for CRPC, *n* (%)	
Androgen receptor pathway inhibitor	162 (75.4%)
Abiraterone	57 (26.5%)
Enzalutamide	105 (48.8%)
Docetaxel	53 (24.7%)

CRPC, castration-resistant prostate cancer; IQR, interquartile range; PSA, prostate-specific antigen.

**Fig. 1:**
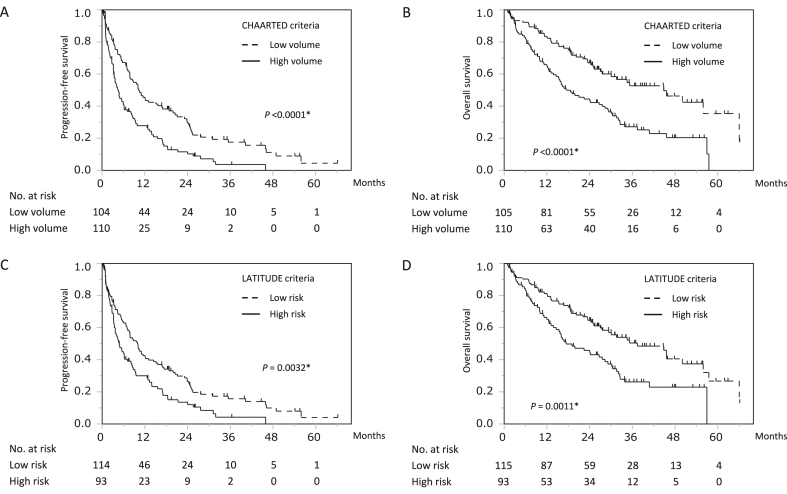
Progression-free survival (PFS) and overall survival (OS) in patients stratified by CHAARTED/LATITUDE criteria. (A) and (B) Kaplan–Meier survival curves of PFS (A) and OS (B) when stratified by CHAARTED criteria. (C) and (D) Kaplan–Meier survival curves of PFS (C) and OS (D) when stratified by LATITUDE criteria.

The median PFS and OS were 7.0 months (95% confidence interval [CI], 5.3–8.8 months) and 28.3 months (95% CI, 22.5–32.5 months), respectively. When patients were divided into two groups according to the CHAARTED criteria, the median PFS was 10.3 months (95% CI, 7.8–15.7 months) in the low-volume group and 4.5 months (95% CI, 3.3–6.2 months) in the high-volume group (Fig. 1A). The median OS was 44.8 months (95% CI, 27.5–66.0 months) in the low-volume group and 17.4 months (95% CI, 13.8–27.5 months) in the high-volume group (Fig. 1B). Similarly, according to the LATITUDE criteria, the median PFS was 9.9 months (95% CI, 7.0–12.2 months) in the low-risk group and 4.8 months (95% CI, 3.4–7.1 months) in the high-risk group (Fig. 1C). The median OS was 37.4 months (95% CI, 27.0–50.1 months) in the low-risk group and 17.4 months (95% CI, 13.8–28.3 months) in the high-risk group (Fig. 1D).

In univariate analysis, higher PSA, > 3 bone metastases, and the use of docetaxel as primary treatment for CRPC, as well as high volume according to the CHAARTED criteria and high risk according to the LATITUDE criteria, were significantly associated with shorter PFS (Table 2). Multivariate analysis showed that both CHAARTED and LATITUDE criteria were significant prognostic factors for PFS (Table 2). In univariate analysis, older age, higher PSA, shorter time to CRPC onset, no prior local treatment, >3 bone metastases, visceral metastasis, and docetaxel as first-line treatment for CRPC, as well as high volume according to the CHAARTED criteria and high risk according to the LATITUDE criteria, were significantly associated with shorter OS (Table 3). In multivariate analysis, the CHAARTED criteria was a significant prognostic factor for OS, while the LATITUDE criteria did not show statistical significance (Table 3).

**Table 2:** Associations between parameters and progression-free survival

Variable	Univariate			Multivariate (CHAARTED)			Multivariate (LATITUDE)		
	HR	95% CI	*P* value	HR	95% CI	*P* value	HR	95% CI	*P* value
Pretreatment age (per 10 years)	1.10	0.91–1.33	0.32	1.27	1.04–1.57	0.022∗	1.27	1.04–1.56	0.021∗
Pretreatment PSA (per 100 ng/mL)	1.02	1.00–1.03	0.0090∗	1.02	1.01–1.03	0.040∗	1.02	1.00–1.04	0.024∗
Time to CRPC (per 12 months)	0.95	0.89–1.01	0.12	0.98	0.92–1.05	0.58	0.98	0.92–1.04	0.48
Gleason score									
≤ 8	ref	–	–	ref	–	–			
> 8	1.06	0.77–1.46	0.71	1.12	0.80–1.55	0.52			
Prior local treatment									
Absence	ref	–	–	ref	–	–	ref	–	–
Presence	0.85	0.62–1.18	0.33	1.22	0.85–1.74	0.28	1.15	0.80–1.64	0.45
Number of bone metastasis									
≤ 3	ref	–	–						
> 3	1.84	1.36–2.49	<0.0001∗						
Viscetal metastasis									
Absence	ref	–	–						
Presence	1.56	0.91–2.65	0.10						
First-line treatment for CRPC									
Androgen receptor pathway inhibitor	ref	–	–	ref	–	–	ref	–	–
Docetaxel	1.90	1.36–2.67	0.0002∗	1.71	1.18–2.48	0.048∗	1.81	1.25–2.62	0.0017∗
CHAARTED criteria									
Low volume	ref	–	–	ref	–	–			
High volume	1.89	1.39–2.57	<0.0001∗	1.78	1.25–2.52	0.0012∗			
LATITUDE criteria									
Low risk	ref	–	–				ref	–	–
High risk	1.58	1.16–2.14	0.0036∗				1.48	1.04–2.10	0.030∗

CI, confidence interval; CRPC, castration-resistant prostate cancer; HR, hazard ratio; PSA, prostate-specific antigen.

∗ Statistically significant.

**Table 3:** Associations between parameters and overall survival

Variable	Univariate			Multivariate (CHAARTED)			Multivariate (LATITUDE)		
	HR	95% CI	*P* value	HR	95% CI	*P* value	HR	95% CI	*P* value
Pretreatment age (per 10 years)	1.40	1.10–1.79	0.0072∗	1.80	1.36–2.38	< 0.0001∗	1.77	1.35–2.34	< 0.0001∗
Pretreatment PSA (per 100 ng/ml)	1.03	1.00–1.05	0.0055∗	1.02	1.00–1.05	0.053	1.03	1.00–1.05	0.033∗
Time to CRPC (per 12 months)	0.89	0.81–0.97	0.014∗	0.96	0.87–1.05	0.38	0.95	0.87–1.04	0.30
Gleason score									
≤ 8	ref	–	–	ref	–	–			
> 8	1.19	0.81–1.76	0.37	1.23	0.82–1.85	0.32			
Prior local treatment									
Absence	ref	–	–	ref	–	–	ref	–	–
Presence	0.57	0.37–0.86	0.0081∗	0.84	0.53–1.33	0.46	0.80	0.50–1.27	0.35
Number of bone metastasis									
≤ 3	ref	–	–						
> 3	2.03	1.41–2.93	0.0001∗						
Viscetal metastasis									
Absence	ref	–	–						
Presence	1.84	1.01–3.36	0.045∗						
First-line treatment for CRPC									
Androgen receptor pathway inhibitor	ref	–	–	ref	–	–	ref	–	–
Docetaxel	2.07	1.41–3.04	0.0002∗	1.98	1.29–3.02	0.0016∗	2.06	1.35–316	0.0008∗
CHAARTED criteria									
Low volume	ref	–	–	ref	–	–			
High volume	2.13	1.47–3.09	<0.0001∗	1.77	1.16–2.71	0.0084∗			
LATITUDE criteria									
Low risk	ref	–	–				ref	–	–
High risk	1.83	1.27–2.65	0.0013∗				1.49	0.97–2.30	0.068

CI, confidence interval; CRPC, castration-resistant prostate cancer; HR, hazard ratio; PSA, prostate-specific antigen.

∗ Statistically significant.

**Fig. 2:**
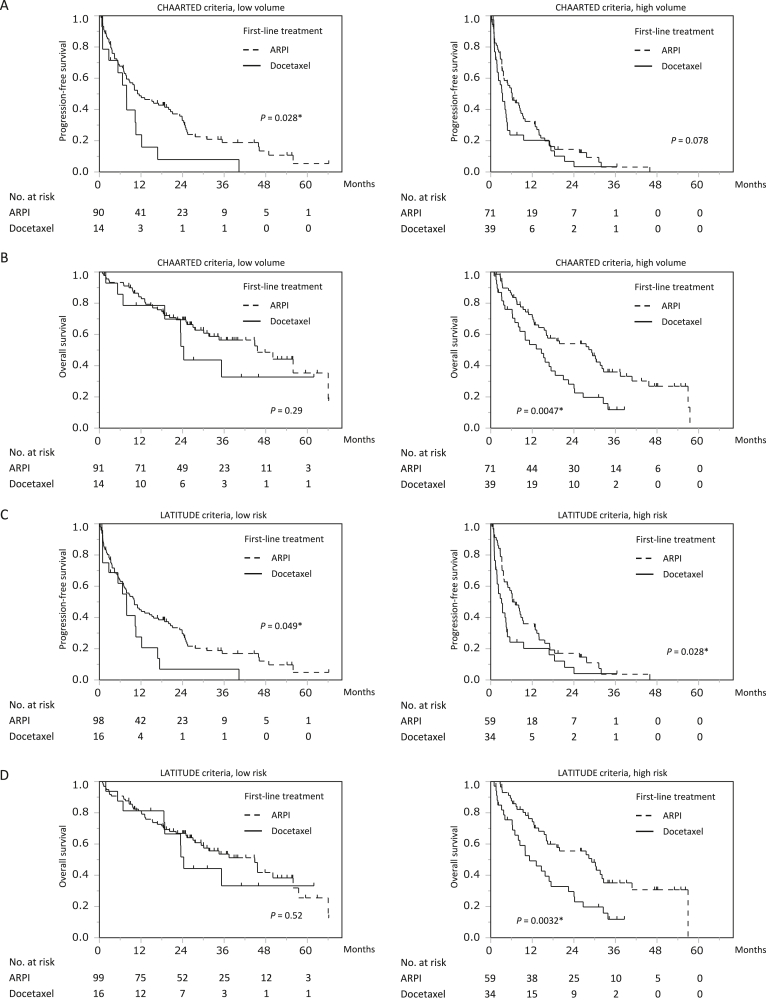
Progression-free survival (PFS) and overall survival (OS) in patients stratified by CHAARTED/LATITUDE criteria and androgen receptor pathway inhibitor (ARPI) or docetaxel as first-line treatment for CRPC. (A) and (B) Kaplan–Meier survival curves of PFS (A) and OS (B) when stratified by CHAARTED criteria and first-line treatment for castration-resistant prostate cancer. (C) and (D) Kaplan–Meier survival curves of PFS (C) and OS (D) when stratified by LATITUDE criteria and first-line treatment for castration-resistant prostate cancer.

We then assessed the prognostic impact of the CHAARTED and LATITUDE criteria by therapeutic agent as primary treatment for CRPC. In the low-volume by the CHAARTED criteria, 91 patients were treated with ARPI and 14 patients with docetaxel while in the high-volume group, 71 patients were treated with ARPI and 39 patients with docetaxel. In low/high volume disease, the median PFS was 11.0/6.2 months (95% CI, 7.8–20.0/3.7–8.7 months) by ARPI and 7.8/3.4 months (95% CI, 0.9–10.5/1.8–4.5 months) by docetaxel (Fig. 2A). In low/high volume disease, median OS was 45.6/29.2 months (95% CI, 30.1–66.0/15.4–32.6 months) by ARPI and 24.3/14.6 months (95% CI, 6.8 months–not reached/7.0–18.6 months) by docetaxel (Fig. 2B).

Similarly, in the low-risk group according to the LATITUDE criteria, 99 patients received ARPI and 16 received docetaxel; in the high-volume group, 59 patients received ARPI and 34 received docetaxel. For low/high risk disease, the median PFS was 10.1/6.3 months (95% CI: 7.0–15.2/3.9–9.4 months) in the ARPI group and 7.8/3.1 months (95% CI: 0.9–12.2/1.8–4.6 months) in the docetaxel group (Fig. 2C). In low/high risk disease, the median OS was 44.8/29.2 months (95% CI, 27.5–55.9/16.0–32.6 months) in the ARPI group and 24.3/11.2 months (95% CI, 18.6 months–not reached/6.3–17.4 months) in the docetaxel group (Fig. 2D).

**Fig. 3:**
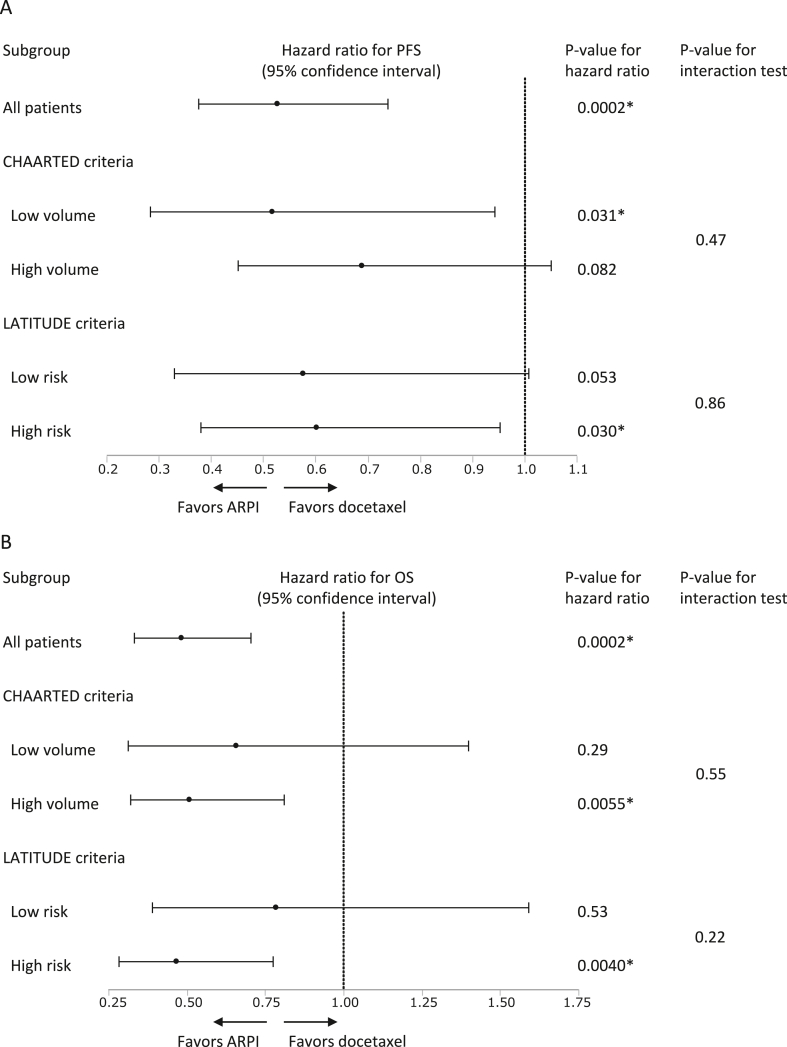
Subgroup analysis on progression-free survival (PFS) and overall survival (OS). (A) and (B) Hazard ratio with 95% confidence interval for PFS (A) and OS (B) in each subgroup when treated with androgen receptor pathway inhibitor (ARPI) or docetaxel. P-values for hazard ratio and interaction test are provided.

Finally, we analyzed the different effects of the CHAARTED and LATITUDE criteria on the clinical outcomes between ARPI and docetaxel. Regarding PFS, the HR favored ARPI over docetaxel in both low- and high-volume diseases by CHAARTED criteria, and both low- and high-risk disease by LATITUDE criteria (Fig. 3A). Similarly, the HR for OS favored ARPI compared to docetaxel in both low- and high-volume diseases by CHAARTED criteria, and both low- and high-risk disease by LATITUDE criteria (Fig. 3B). Consistently, P-value for interaction test showed no statistical significance, indicating the CHAARTED and LATITUDE criteria did not discriminate therapeutic effect between ARPI and docetaxel.

## 4 Discussion

In metastatic HSPC, CHAARTED and LATITUDE criteria have shown excellent risk stratification.^9^^,^^11^^,^^17^, ^18^, ^19^, ^20^ Our study showed that both criteria at the diagnosis of CRPC were prognostic factors, suggesting that these prognostic criteria apply to CRPC. Interestingly, the CHAARTED criteria was shown to be an independent prognostic factor for both PFS and OS, while the LATITUDE criteria was shown to be an independent prognostic factor only for PFS. As shown in this study, biopsy Gleason score at initial diagnosis is not a prognostic factor when disease progressed to CRPC. This difference may be derived from the fact that the LATITUDE criteria used Gleason score as one of 3 risk parameters. Taken together, these findings suggested that the CHAARTED criteria is more suitable as a prognostic factor in CRPC compared to the LATITUDE criteria.

So far, several risk classifications in CRPC have been reported.^21^ Armstrong et al. used data from the TAX327 trial to construct a risk model consisting of 11 factors that predicted OS in men treated with docetaxel chemotherapy.^22^ Halabi et al. used data from the TROPIC trial to create a nine-parameter risk model for patients who received second-line chemotherapy.^23^ In addition, Chi et al. proposed a risk model with 6 predictors in patients treated with abiraterone acetate after docetaxel using the COU-AA-301 trial.^24^ In those risk models, parameters such as pain, performance status, serum markers (PSA, hemoglobin, alkaline phosphatase, lactate dehydrogenase, and albumin), tumor grade, metastatic sites, and disease kinetics were utilized. Although these risk classification models have been validated by different cohorts, many parameters to estimate the risk are required in these models.^25^, ^26^, ^27^ The advantage of the LATITUDE and CHAARTED criteria is that they require relatively few factors for evaluation. Also, these criteria showed consistent prognostic values when treated with ARPI and docetaxel as first-line treatments for CRPC.

Currently, ARPI and docetaxel are recommended first-line treatments for CRPC. Yamamoto et al. compared docetaxel and ARPI as first-line treatments for CRPC by propensity-score matching.^28^ They analyzed 234 patients and reported that ARPI had a longer OS for CRPC patients compared to docetaxel. On the other hand, Sonpavde et al. similarly examined OS with docetaxel and ARPI as first-line therapy in 1445 patients with metastatic CRPC, and found OS was similar for first-line chemotherapy compared to ARPI.^29^ Thus, there is no consensus on the superiority of first-line treatment for CRPC. Then, a biomarker to choose ARPI and docetaxel is needed. In HSPC, the CHAARTED criteria have been suggested to be a useful factor in choosing ARPI and docetaxel.^30^ Accordingly, this study investigated whether there is a difference in the therapeutic effect of ARPI and docetaxel in two groups divided by the CHAARTED and LATITUDE criteria, and found no differential impact of the CHAARTED and LATITUDE criteria in treatment with ARPI or docetaxel chemotherapy. Therefore, further investigations are warranted to determine a useful biomarker for treatment selection.

This study has several limitations. The design was retrospective and the sample size was small. It was up to the physician's discretion to decide whether to use docetaxel or ARPI after the diagnosis of CRPC, which may lead to bias. In addition, the second and subsequent treatments were not defined, and it may have affected the OS. These limit our ability to draw definitive conclusions.

For the first time, this study indicated that risk stratification by the LATITUDE as well as CHAARTED criteria in CRPC is prognostic of disease progression and OS. However, those criteria were not useful in choosing treatment using ARPI or docetaxel.

## Grant support

None.

## Conflicts of interest

Masaki Shiota, Akira Yokomizo, and Masatoshi Eto have received honoraria from Janssen Pharma, Astellas Pharma, AstraZeneca, Takeda Pharmaceutical, and Sanofi.

## References

[1] Shiota M., Eto M. (2016). Current status of primary pharmacotherapy and future perspectives toward upfront therapy for metastatic hormone-sensitive prostate cancer. Int J Urol.

[2] Choi S.Y., Ryu J., You D., Hong J.H., Ahn H., Kim C.S. (2019). Simple risk assessment in prostate cancer patients treated with primary androgen deprivation therapy: The Korean Cancer Study of the Prostate risk classification. Int J Urol.

[3] Tannock I.F., de Wit R., Berry W.R., Horti J., Pluzanska A., Chi K.N. (2004). Docetaxel plus prednisone or mitoxantrone plus prednisone for advanced prostate cancer. N Engl J Med.

[4] de Bono J.S., Logothetis C.J., Molina A., Fizazi K., North S., Chu L. (2011). Abiraterone and increased survival in metastatic prostate cancer. N Engl J Med.

[5] Ryan C.J., Smith M.R., de Bono J.S., Molina A., Logothetis C.J., de Souza P. (2013). Abiraterone in metastatic prostate cancer without previous chemotherapy. N Engl J Med.

[6] Scher H.I., Fizazi K., Saad F., Taplin M.E., Sternberg C.N., Miller K. (2012). Increased survival with enzalutamide in prostate cancer after chemotherapy. N Engl J Med.

[7] Beer T.M., Armstrong A.J., Rathkopf D.E., Loriot Y., Sternberg C.N., Higano C.S. (2014). Enzalutamide in metastatic prostate cancer before chemotherapy. N Engl J Med.

[8] Nuhn P., De Bono J.S., Fizazi K., Freedland S.J., Grilli M., Kantoff P.W. (2019). Update on Systemic Prostate Cancer Therapies: Management of Metastatic Castration-resistant Prostate Cancer in the Era of Precision Oncology. Eur Urol.

[9] Kyriakopoulos C.E., Chen Y.H., Carducci M.A., Liu G., Jarrard D.F., Hahn N.M. (2018). Chemohormonal therapy in metastatic hormone-sensitive prostate cancer: long-term survival analysis of the randomized phase III E3805 CHAARTED trial. J Clin Oncol.

[10] Fizazi K., Tran N., Fein L., Matsubara N., Rodriguez-Antolin A., Alekseev B.Y. (2019). Abiraterone acetate plus prednisone in patients with newly diagnosed high-risk metastatic castration-sensitive prostate cancer (LATITUDE): final overall survival analysis of a randomised, double-blind, phase 3 trial. Lancet Oncol.

[11] Shiota M., Namitome R., Kobayashi T., Inokuchi J., Tatsugami K., Eto M. (2019). Prognostic significance of risk stratification in CHAARTED and LATITUDE studies among Japanese men with de novo metastatic prostate cancer. Int J Urol.

[12] Hatakeyama S., Narita S., Takahashi M., Sakurai T., Kawamura S., Hoshi S. (2020). Association of tumor burden with the eligibility of upfront intensification therapy in metastatic castration-sensitive prostate cancer: A multicenter retrospective study. Int J Urol.

[13] Sobin L.H., Wittekind C.H., International Union Against Cancer (1997). TNM Classification of Malignant Tumors.

[14] Scher H.I., Halabi S., Tannock I., Morris M., Sternberg C.N., Carducci M.A. (2008). Design and end points of clinical trials for patients with progressive prostate cancer and castrate levels of testosterone: recommendations of the Prostate Cancer Clinical Trials Working Group. J Clin Oncol.

[15] Shiota M., Yokomizo A., Adachi T., Koga H., Yamaguchi A., Imada K. (2014). The oncological outcomes and risk stratification in docetaxel chemotherapy for castration-resistant prostate cancer. Jpn J Clin Oncol.

[16] Shiota M., Yokomizo A., Takeuchi A., Kiyoshima K., Inokuchi J., Tatsugami K. (2016). Co-introduction of a steroid with docetaxel chemotherapy for metastatic castration-resistant prostate cancer affects PSA flare. BJU Int.

[17] Francini E., Gray K.P., Xie W., Shaw G.K., Valença L., Bernard B. (2018). Time of metastatic disease presentation and volume of disease are prognostic for metastatic hormone sensitive prostate cancer (mHSPC). Prostate.

[18] Buelens S., Poelaert F., Dhondt B., Fonteyne V., De Visschere P., Ost P. (2018). Metastatic burden in newly diagnosed hormone-naive metastatic prostate cancer: Comparing definitions of CHAARTED and LATITUDE trial. Urol Oncol.

[19] Gravis G., Boher J.M., Chen Y.H., Liu G., Fizazi K., Carducci M.A. (2018). Burden of Metastatic Castrate Naive Prostate Cancer Patients, to Identify Men More Likely to Benefit from Early Docetaxel: Further Analyses of CHAARTED and GETUG-AFU15 Studies. Eur Urol.

[20] Kawahara T., Yoneyama S., Ohno Y., Iizuka J., Hashimoto Y., Tsumura H. (2020). Prognostic value of the LATITUDE and CHAARTED risk Criteria for predicting the survival of men with bone metastatic hormone-naïve prostate cancer treated with combined androgen blockade therapy: real-world data from a Japanese multi-institutional study. Biomed Res Int.

[21] Pinart M., Kunath F., Lieb V., Tsaur I., Wullich B., Schmidt S. (2020). German Prostate Cancer Consortium (DPKK). Prognostic models for predicting overall survival in metastatic castration-resistant prostate cancer: a systematic review. World J Urol.

[22] Armstrong A.J., Garrett-Mayer E.S., Yang Y.C., de Wit R., Tannock I.F., Eisenberger M. (2007). A contemporary prognostic nomogram for men with hormone-refractory metastatic prostate cancer: a TAX327 study analysis. Clin Cancer Res.

[23] Halabi S., Lin C.Y., Small E.J., Armstrong A.J., Kaplan E.B., Petrylak D. (2013). Prognostic model predicting metastatic castration-resistant prostate cancer survival in men treated with second-line chemotherapy. J Natl Cancer Inst.

[24] Chi K.N., Kheoh T., Ryan C.J., Molina A., Bellmunt J., Vogelzang N.J. (2016). A prognostic index model for predicting overall survival in patients with metastatic castration-resistant prostate cancer treated with abiraterone acetate after docetaxel. Ann Oncol.

[25] Kawahara T., Miyoshi Y., Sekiguchi Z., Sano F., Hayashi N., Teranishi J. (2012). Risk factors for metastatic castration-resistant prostate cancer (CRPC) predict long-term treatment with docetaxel. PLoS One.

[26] Nakano K., Komatsu K., Kubo T., Natsui S., Nukui A., Kurokawa S. (2014). External validation of risk classification in patients with docetaxel-treated castration-resistant prostate cancer. BMC Urol.

[27] Yang Y.J., Lin G.W., Li G.X., Dai B., Ye D.W., Wu J.L. (2018). External validation and newly development of a nomogram to predict overall survival of abiraterone-treated, castration-resistant patients with metastatic prostate cancer. Asian J Androl.

[28] Yamamoto A., Kato M., Hattori K., Naito Y., Tochigi K., Sano T. (2020). Propensity score-matched comparison of docetaxel and androgen receptor axis-targeted agents in patients with castration-resistant intraductal carcinoma of the prostate. BJU Int.

[29] Sonpavde G., Huang A., Wang L., Baser O., Miao R. (2018). Taxane chemotherapy vs antiandrogen agents as first-line therapy for metastatic castration-resistant prostate cancer. BJU Int.

[30] James N.D. (2019). Oligometastatic prostate cancer should be studied and treated differently to high-volume disease. Con: the underlying biology is the same, so they should not be treated differently. Eur Urol Focus.

